# Evaluating next-generation sequencing for direct clinical diagnostics in diarrhoeal disease

**DOI:** 10.1007/s10096-017-2947-2

**Published:** 2017-03-11

**Authors:** K. G. Joensen, A. L. Ø. Engsbro, O. Lukjancenko, R. S. Kaas, O. Lund, H. Westh, F. M. Aarestrup

**Affiliations:** 1 National Food Institute, Division for Epidemiology and Microbial Genomics, Technical University of Denmark, Søltofts Plads, Building 221, 2800 Kgs. Lyngby, Denmark; 2 Department of Microbiology and Infection Control, Statens Serum Institute, Copenhagen, Denmark; 3 Department of Clinical Microbiology, Hvidovre University Hospital, Copenhagen, Denmark; 4 Department of Medical Gastroenterology, Køge University Hospital, Køge, Denmark; 5 Center for Biological Sequence Analysis, Department of System Biology, Technical University of Denmark, Kgs. Lyngby, Denmark; 6 Faculty of Health Sciences, University of Copenhagen, Copenhagen, Denmark

## Abstract

The accurate microbiological diagnosis of diarrhoea involves numerous laboratory tests and, often, the pathogen is not identified in time to guide clinical management. With next-generation sequencing (NGS) becoming cheaper, it has huge potential in routine diagnostics. The aim of this study was to evaluate the potential of NGS-based diagnostics through direct sequencing of faecal samples. Fifty-eight clinical faecal samples were obtained from patients with diarrhoea as part of the routine diagnostics at Hvidovre University Hospital, Denmark. Ten samples from healthy individuals were also included. DNA was extracted from faecal samples and sequenced on the Illumina MiSeq system. Species distribution was determined with MGmapper and NGS-based diagnostic prediction was performed based on the relative abundance of pathogenic bacteria and *Giardia* and detection of pathogen-specific virulence genes. NGS-based diagnostic results were compared to conventional findings for 55 of the diarrhoeal samples; 38 conventionally positive for bacterial pathogens, two positive for *Giardia*, four positive for virus and 11 conventionally negative. The NGS-based approach enabled detection of the same bacterial pathogens as the classical approach in 34 of the 38 conventionally positive bacterial samples and predicted the responsible pathogens in five of the 11 conventionally negative samples. Overall, the NGS-based approach enabled pathogen detection comparable to conventional diagnostics and the approach has potential to be extended for the detection of all pathogens. At present, however, this approach is too expensive and time-consuming for routine diagnostics.

**Electronic supplementary material:**

The online version of this article (doi:10.1007/s10096-017-2947-2) contains supplementary material, which is available to authorized users.

## Introduction

Diarrhoea has a global disease burden estimated to encompass 1.7 billion cases each year, with 1.5 million deaths worldwide attributed in 2012, and is the second most common cause of death in young children [1, 2]. Diarrhoea is typically a symptom of a gastrointestinal infection, but may also be a symptom of several medical conditions or a result of drug treatment, e.g. antibiotic-associated diarrhoea [3].

Diarrhoea of different infectious origin (bacterial, viral and some parasitic) may be difficult to distinguish based on history or clinical observations and, thus, rapid laboratory analyses are important, since treatment and patient care depends on the pathogen [1, 4, 5]. In addition, rapid and accurate diagnostics, characterisation and comparison of pathogens are essential to identify both nosocomial and foodborne outbreaks. However, diagnostic results are often not available in a timely fashion and current methods employed are labourious, time-consuming, costly, require significant expertise and result in the detection of pathogens in only a small fraction of examined samples [4, 5]. Thus, conventional diagnostics typically only result in the identification of a minority of diarrhoea-causing microbial agents, while up to 80% of cases remain unresolved [6]. It is also well -known that some bacterial pathogens are difficult to grow or are even non-culturable, while still being viable [7].

Polymerase chain reaction (PCR)-based methods for the detection of enteropathogens from stool samples that are more rapid and more sensitive than the conventional culturing procedures have been described [8–11]. The disadvantage of using PCR may be that we are only detecting those agents we are looking for and it is normally not possible to obtain phylogenetic information.

Next-generation sequencing (NGS) has started to gain ground in public health and clinical microbiology. NGS provides cost-efficient analysis and rapid turnaround time [12, 13]. It has already been used in clinical settings for elucidating bacterial outbreaks [14–16] and it has been proposed for the real-time typing and surveillance of pathogens [16–18].

NGS has, until recently, been employed mainly on bacterial isolates. However, as demonstrated for urinary tract infections [19], the technology can be applied directly to clinical samples, potentially advancing diagnostics and leading to even more rapid diagnostic results. Furthermore, it was recently demonstrated that the detection of *Clostridium difficile* by NGS is correlated with the already existing laboratory testing [20] and metagenomics sequencing has been employed on a limited number of patient stool samples for the detection of pathogens [21, 22]. In addition to species detection, NGS offers detection of resistance and virulence genes, which can further shorten the time needed for pathogen-directed treatment to be initiated.

Here, we evaluated the use of NGS for the diagnostics of diarrhoea by comparing direct NGS sequencing of human faecal samples to the outcome of the conventional diagnostic procedures on several bacterial pathogens.

## Materials and methods

### Faecal samples

Clinical faecal samples were included based on positive bacterial, viral or parasitological findings at the Department of Clinical Microbiology at Hvidovre University Hospital in Denmark. We included samples positive for *C. difficile* (*n* = ∼15), *Salmonella* spp. (*n* = 4–6), *Campylobacter* spp. (*n* = 2–4), *Yersinia enterocolitica* (*n* = 0–2), diarrhoeagenic *Escherichia coli* (*n* = ∼10), *Giardia intestinalis* (*n* = ∼3), rotavirus (*n* = ∼4) and norovirus (*n* = ∼4), as well as samples (*n* = ∼15) obtained from diarrhoeal patients, with no microbiological findings in the routine diagnostics. The samples were collected from September to November 2013. As controls, we included faecal samples from healthy adults participating in a clinical randomised trial on the interactions between antibiotics and the gut microbiota conducted at Køge Hospital, Denmark (local ethics committee journal number SJ-383). Control samples were collected during June 2014 prior to antibiotic treatment.

Faeces were stored either as whole faeces in 1 mL STAR buffer or as the liquid solution from a FaecalSwab (Copan), depending on how the sample had been submitted to the Department of Clinical Microbiology for analysis. Bacterial pathogens cultured from the samples were stored in broth with 10% glycerol. All faecal samples and isolates were stored at −80 °C. For all included patients and healthy study participants, age and gender were registered, and for patients, the information on occurrence of bloody diarrhoea was noted as reported on the sample requisition form.

### Conventional routine laboratory analysis

Routine conventional diagnostic tests were performed on all samples and included conventional bacterial culturing for enteropathogens as well as PCR for diarrhoeagenic *E. coli* (DEC), toxigenic *C. difficile*, diarrhoeagenic viruses and intestinal parasites. DEC PCR was conducted on the isolated *E. coli* by in-house conventional multiplex PCR, detecting A/EEC: intimin (*eae*); EPEC: A/EEC (*eae*) with classical EPEC serotype (O26, O103, O111, O145, O157, O55, O119, O125ac, O127, O128ab, O86, O114, O121, O126 or O142); VTEC: verocytotoxin 1 and 2 (*vtx1*, *vtx2*); ETEC: heat-stable (ST) enterotoxin (*estA*) and heat-labile (LT) enterotoxin (*eltA*); EIEC: invasive plasmid antigen (*ipaH*); and EAEC: transcriptional activator (*aggR*) [23].

For *C. difficile*, real-time PCR was conducted directly on faecal samples according to an in-house protocol for the detection of toxin A (*tcdA*), toxin B (*tcdB*), binary toxin (*cdtA*) and for mutation (Δ117) in the regulator *tcdC* [24].

The diarrhoeagenic viruses were examined directly on faecal samples by an in-house real-time PCR covering norovirus (genotypes I and II), sapovirus (genotypes I, II and V), adenovirus F (serotypes 40 and 41) and rotavirus (A and C).

The presence of intestinal parasites was examined by stool microscopy and additionally by a multiplex real-time PCR assay employing the LightMix Modular Assay (TIB MOLBIOL GmbH, Germany) for *Entamoeba histolytica*, *G. intestinalis*, *Cryptosporidium* sp. and *Dientamoeba fragilis*.

### DNA isolation and sequencing

DNA was isolated from the entire amount of stored faecal samples using the QIAamp DNA Stool Kit (Qiagen, Denmark) for the isolation of DNA for pathogen detection with slight modifications. In the initial lysis step (3), an additional 5 min of incubation at 95 °C was performed and in the final step (18), elution was done with 100 μL Buffer AE. DNA purification from bacterial isolates was done employing the Easy-DNA Kit (Invitrogen, Denmark), according to the manufacturer’s protocol. The DNA concentrations were measured with the Qubit® dsDNA HS and Qubit® dsDNA BR Assay Kits (Invitrogen) and DNA libraries were constructed using the Nextera XT DNA Sample Preparation Kit (Illumina, Denmark), according to the manufacturer’s protocol. Sequencing was performed on the MiSeq system (Illumina), employing the MiSeq Reagent Kit v2 (Illumina) for 500 cycles (2 × 250 bp) for patient samples and bacterial isolates, whereas control samples were sequenced employing the MiSeq Reagent Kit v3 (Illumina) for 600 cycles (2 × 300 bp). Four samples were sequenced per MiSeq run.

Five of the samples (S_127, S_164, H_107, H_108 and H_110) were sequenced from both purification of faeces and faecal swabs to ensure comparability.

All sequences of metagenomic samples, with human DNA removed, as well as sequences of isolates, have been deposited at ENA (PRJEB14038).

### Analysis of sequencing results

(i) Species confirmation and virulence profiling of isolate sequences
  The isolate sequences included in the study were subjected to species identification employing KmerFinder 1.3 [19, 25]. Additional information on this method is included in Supplemental File 1. All isolate sequences were examined for the presence of selected virulence genes for *E. coli* (*aggR*, *astA*, *eae*, *eltA*, *eltB*, *estA*, *stx1A*, *stx1B*, *stx2A*, *stx2B*), *Shigella* spp. (*ipaB*, *ipaC*, *ipaD*, *ipaH7.8*, *ipaH9.8*, *mxiA*, *stxA*, *stxB*, *virA*), *C. jejuni* (*cdtA*, *cdtB*, *cdtC*, *ciaB*, *flaA*, *flaB*, *flaC*), *C. difficile* (*cdtA*, *cdtB*, *tcdA*, *tcdB*), *S. enterica* (*invA*, *invB*, *invE*, *invG*, *invH*, *invJ*, *sseA*, *sseB*, *sseC*, *sseD*, *sseE*) and *Y. enterocolitica* (*inv*, *ystA*) using MGmapper (https://cge.cbs.dtu.dk/services/MGmapper), mapping against the Virulence Factors Database (http://www.mgc.ac.cn/VFs/) [26, 27]. For the available isolates, the proportion of the genome sequence within the corresponding faecal sample was determined.
(ii) Metagenomic analysis and species distribution in faecal samples
  The distribution of species within the faecal samples was determined using MGmapper (https://cge.cbs.dtu.dk/services/MGmapper/) [26]. Specifically, paired-end reads from each metagenome sample were mapped against six databases: human genomes, parasite genomes, complete bacterial genomes, as well as draft bacterial genomes, obtained from GenBank (http://www.ncbi.nlm.nih.gov/genbank/), Virulence Factor (http://www.mgc.ac.cn/VFs/) [28] and VirulenceFinder [17]. Additional information on this method is included in Supplemental File 1.
  The composition of organisms was evaluated by the number of reads mapping to all individual organisms. The relative abundance of human, bacterial and parasitic DNA, as well as that of defined pathogens (*S. enterica*, *Y. enterocolitica*, *E. coli*, *C. jejuni*, *C. difficile*, *Shigella* and *G. intestinalis*) was calculated as the percentage of reads (of total reads in the sample) mapping to the particular pathogen or group (human, bacterial or parasitic). For each of the specific pathogens, the relative abundance of the pathogen was determined for: (1) patients with diarrhoea caused by the particular pathogen, (2) patients with diarrhoea where the particular pathogen was not detected by conventional methods and (3) healthy controls, all according to conventional diagnostics. Similarly, the relative abundance of pathogens was also examined for the conventionally negative samples and the samples where only viral pathogens were determined by conventional diagnostics. Additionally, for each of the specific pathogens, the ratio between the relative abundance of the pathogen and Shannon’s diversity index of the sample was determined.
  Differences in relative abundance between samples collected as faeces and swabs were assessed by comparing the relative abundance within the samples of all detected hits, on the species level, covering all employed MGmapper databases. Interquartile ranges (IQRs) and upper fence values were calculated for each of the pathogens of interest in both control samples and diarrhoea samples from other pathogen origin. The size of the IQR (Q_3_ − Q_1_) indicates how spread the middle half of the data is. Q_3_ + 1.5×IQR was used as a measure (the upper fence) to identify values that were much farther away from the centre (outliers). An NGS-based case diagnosis was assigned to a sample if: (1) the relative abundance of the pathogen was higher than the upper fence (Q_3_ + 1.5×IQR) of the control samples and also higher than the upper fence (Q_3_ + 1.5×IQR) of diarrhoea samples from other pathogen origin (together referred as the threshold) or (2) pathogen-specific virulence factors were detected.
(iii) Typing of *E. coli* and *C. difficile* by direct sequencing
  To evaluate the possibility of performing bacterial typing by direct sequencing, samples conventionally positive for *E. coli* or *C. difficile* and the corresponding isolates were subjected to phylogenetic analysis employing the tool NDtree, which infers phylogenies based on the number of nucleotide differences between isolates. Phylogenies were inferred for *E. coli* and *C. difficile*, employing the reference strains *E. coli* O157:H7 str. Sakai (accession number NC_002695.1) and *Peptoclostridium difficile* 630 (accession number NC_009089.1), respectively. NDtree was employed as described by Joensen et al. [17], with the z-score parameter set to 1.96 and the mode set to pairwise comparison.

## Results

### Collected faeces samples

In total, 58 patient samples and ten samples from healthy individuals were collected and included in the study (Table 1). In 45 of the patient samples, one or more pathogens had been identified by the routine diagnostics: 39 with bacteria, nine with viruses and two with parasites. Some samples contained more than one pathogen: seven different bacterial species and three samples had multiple DEC. Five samples contained both bacterial and viral pathogens. Thirteen patient samples were initially included, which were negative according to the conventional diagnostic tests. The median age of the patients was 47.5 years (range 0–95 years) and 36 patients (62%) were female. In the control group, the median age was 38 years (range 23–56 years) and 60% were female. Nine patients had bloody diarrhoea, 33 had non-bloody diarrhoea and for 13 patients, no information was available.

**Table 1:** Findings in faecal samples by conventional diagnostic methods

Pathogen	Number of samples	Sample ID
Bacterial		
*Clostridium difficile*	14^a^ (13)	S_105, S_106, S_107, S_108 ^a^, **S_130**, S_134, S_136, S_137, **S_149**, S_160^B^, S_162, **S_164**, S_165, S_166
*Salmonella enterica* subsp. *enterica*	5	**S_126**, S_127, S_128, S_129, **S_153** ^B^
*Shigella* spp.	2	S_144^B^, S_152^B^
*Campylobacter jejuni*	5	S_102^B^, **S_103**, S_104, S_132^B^, S_138^B^
*Yersina enterocolitica*	2	S_143, **S_164**
DEC-positive *Escherichia coli*	15	S_110*, **S_130**, **S_132** ^B^, **S_140**, S_141, S_142, S_145, **S_148**, S_151, **S_153** ^B^, S_154*, S_156*, S_157, S_158, S_159
DEC-positive *E. albertii*	1	**S_148**
*Aeromonas* spp.	1	**S_126**
*Providencia alcalifaciens*	1	**S_140**
Viral		
Sapovirus	2	**S_130**, S_131
Norovirus	5	**S_126**, S_133, S_135, **S_149**, S_150
Rotavirus	1	**S_148**
Adenovirus	2	**S_132** ^B^, **S_148**
Parasitic		
*Giardia intestinalis*	2	S_111, S_ 155
Negative	13^a^ (11)	S_114^B^, S_115, S_116 ^a^, S_117 ^aB^, S_118, S_119, S_120, S_121, S_122, S_123, S_124, S_161^B^, S_163
Healthy controls	10	H_101, H_102, H_103, H_104, H_105, H_106, H_107, H_108, H_110, H_111

Samples where more than one pathogen was detected by conventional diagnostics are in **bold**. Samples from patients with bloody diarrhoea are marked with superscript ^B^. Non-primary pathogens are underlined. An asterisk (*) denotes samples where two different DECs were isolated from conventional diagnostics

^a^Three samples (S_108, S_116 and S_117) were collected for the study but not included in the final analysis since not enough DNA could be purified from the samples

DNA was purified from all 58 patient faecal samples except for three with too low levels of DNA for detection. Two of these samples were conventionally negative (S_116 and S_117) and one was *C. difficile*-positive (S_108), and, thus, further analysis was performed on 55 samples. The DNA purifications ranged in concentrations from 0.12 to 50.2 μg/μL, with the DNA isolations from pure faeces giving the highest yields. The raw sequence output from the patient samples ranged from 125.05 Mb (sample S_136) to 4.26 Gb (sample S_160) and between 1.53 Gb (H_102) and 6.93 Gb (H_111) for healthy controls.

### Abundance of pathogens in faeces samples

**Fig. 1:**
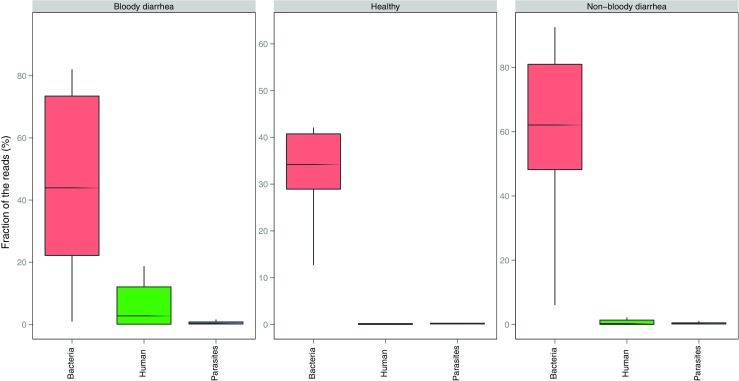
Abundance of bacterial, parasitic and human DNA among faecal samples. For each group of samples, healthy, patients with bloody diarrhoea and patients with non-bloody diarrhoea (or unknown), the fraction of reads mapping to bacteria, parasites and human reference genomes is shown. The abundance is normalised according to the total number of reads in each specific sample

Figure 1 shows the relative abundance of bacterial, human and parasitic DNA for all samples. The relative abundance of bacteria ranged between 46 and 71% for the samples from healthy controls and between 6 and 96% for patient samples. Human DNA content ranged from 0 to 1% in healthy control samples and from 0 to 91% for patient samples. The relative abundance for parasite DNA was low, with only a few sequence reads mapping to parasite sequences for both healthy controls and patients. A large proportion of reads did not map to any sequences present in the databases.

The sampling method employed was not found to have an effect on the relative abundance of species detected in the samples, as evaluated on five samples with DNA extracted from both pure faeces and faecal swabs.

**Fig. 2:**
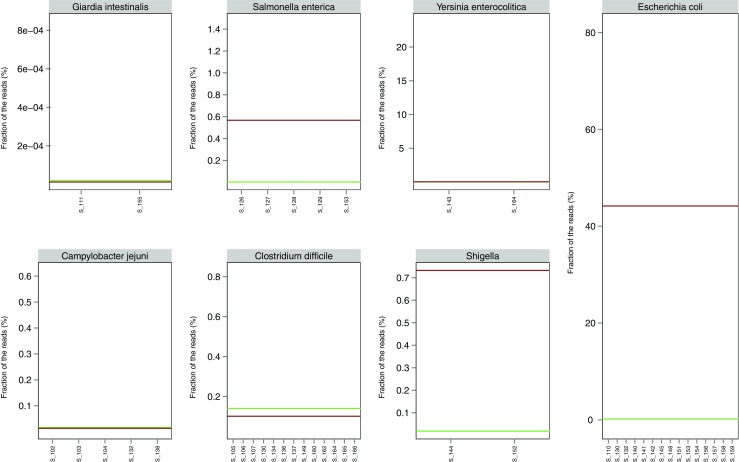
Relative abundance of pathogens in samples positive by conventional diagnostics. For each pathogen (*Giardia*, *Salmonella*, *Y. enterocolitica*, *E. coli*, *C. jejuni*, *C. difficile* and *Shigella*), the fraction of reads mapping to the pathogen is plotted for all samples positive by conventional diagnostic methods. The *orange dots* indicate the presence of pathogen-specific virulence genes as determined by NGS analysis, while the *green dots* indicate the absence. The upper fence (Q_3_ + 1.5×IQR) of the relative abundance for the healthy controls and for the diarrhoea samples where the particular pathogen was not detected by conventional methods are shown

Figure 2 shows the relative abundance of *Giardia*, *Salmonella*, *Y. enterocolitica*, *E. coli*, *C. jejuni*, *C. difficile* and *Shigella* for samples positive for the particular pathogen by conventional diagnostics, compared to healthy controls (see variation of controls in Supplemental Fig. 1) and to the level detected in diarrhoeal samples of other pathogenic origin. The abundance upper fence of the different pathogens varied, with the upper fence of diarrhoea samples from other pathogen origin ranging from 0.00% for *G. intestinalis* to 44.18% for *E. coli*, with *Campylobacter* (0.01%), *Y. enterocolitica* (0.03%), *C. difficile* (0.10%), *Salmonella* (0.57%) and *Shigella* (0.73%) in between. In the healthy controls, the upper fences were: *Giardia* (0.00%), *E. coli* (0.16%), *C. jejuni* (0.02%), *Y. enterocolitica* (0.00%), *C. difficile* (0.14%), *S. enterica* (0.00%) and *Shigella* (0.02%).

For *S. enterica*, all five conventionally positive samples were above the abundance upper fence in the healthy control samples. However, only three were also above the upper fence detected in other non-*Salmonella* diarrhoea samples and, therefore, above the threshold set for NGS-based diagnostics on the basis of relative abundance. These three samples contained detectable *Salmonella* virulence genes (*invA*, *invB*, *invE*, *invG*, *invH*, *invJ*, *sseB*, *sseC*, *sseE*), while one of the *Salmonella*-positive samples (S_127) below the threshold also contained two reads mapping to *invG*. In the last conventionally positive sample (S_129), neither relative abundance nor virulence genes indicated the presence of *Salmonella*. *Salmonella* virulence genes were detected in one sample (S_119) from a diarrhoea patient considered negative by all the conventional diagnostic tests.

For *C. difficile*, seven of the 13 conventionally positive samples were above the upper fence for non-*C. difficile* diarrhoeal samples, but above the level of the healthy controls. In ten of these, sequence reads mapping to *C. difficile* virulence genes (*cdtA*, *cdtB*, tcdA, *tcdB*) were detected. *Clostridium difficile* virulence genes were not detected among other samples except one *Salmonella*-positive sample (S_128), in which two reads mapping to *cdtB* were found. The 15 conventionally DEC-positive *E. coli* samples varied considerably in their relative abundance of *E. coli* sequence (from 0.7 to 80%), and although the abundance was higher than the upper fence for the healthy controls, all but one of the samples were below the level of *E. coli* detected in non-*E. coli* diarrhoeal samples. However, sequences of *E. coli* virulence genes (*stx1A*, *stx1B*, *eae*, *astA*, *eltA*, *eltB*, *aggR*, *estA*) were detected in 14 of the 15 samples, excluding S_158. The *E. coli* virulence genes were generally not found in other samples. However, a few *astA* reads were detected in three samples of other pathogen origin (S_104, S_107, S_129) and in one healthy control (H_108), while the *eae* gene was detected in three samples not found to be *E. coli*-positive by conventional diagnostics (S_127, S_135, S_163).

Both *Shigella*-positive samples were below the upper fence defining the non-*Shigella* diarrhoeal samples, and above the level in the healthy controls. In both samples, however, *Shigella* virulence genes (*virA*, *mxiA*, *ipaH7.8*, *ipaH9.8*, *ipaB*, *ipaD*) were detected. *Shigella* virulence genes were not detected in any other faecal samples. For the five samples which tested positive for *C. jejuni* in conventional diagnostics, three had a relative abundance above the upper fence of both the control group and non-*C. jejuni* diarrhoeal samples. However, for all five samples, *Campylobacter* virulence genes (*ciaB*, *flaA*, *flaB*, *flaC*, *cdtA*, *cdtB*, *cdtC*) were detected. Few *Campylobacter* virulence genes were detected in five samples that were not found to be *Campylobacter*-positive by conventional diagnostics: one positive for norovirus (S_135), three negative by conventional diagnostics (S_114, S_118, S_119) and one a healthy control (H_111).

For the two samples positive for *Y. enterocolitica*, the relative abundance was very different. One sample (S_143) contained around 0.5% *Y. enterocolitica*, which was just above the upper fence for the other diarrhoeal samples, while the other (S_164) had more than 23% *Y. enterocolitica*. Sequence reads mapping to virulence genes (*inv*, *ystB*) were detected in both samples and *Yersinia* virulence genes were not detected in any other samples. Both *G. intestinalis*-positive samples were very low in abundance (below 0.01%), containing only a few reads mapping to *Giardia*. None of the other diarrhoeal samples contained *Giardia* sequences, whereas two healthy controls (H_103, H_107) contained a few reads.

The complete distribution of bacteria within the samples is included in Supplemental Fig. 2.

Similar detection of the pathogens in the faecal samples was performed using the relative abundance of the pathogen with the respect to the Shannon’s diversity index of the sample. The results are comparable with pathogen detection, described above, and are shown in Supplemental Fig. 3. Using this measure, the *S. enterica* sample S_126 moved below the upper fence detected in other non-*Salmonella* diarrhoea samples. However, two *E. coli* samples (S_140, S_153) appeared above the upper fence detected in other non-*Escherichia* diarrhoea samples.

### Detection of pathogens in conventionally negative samples

**Fig. 3:**
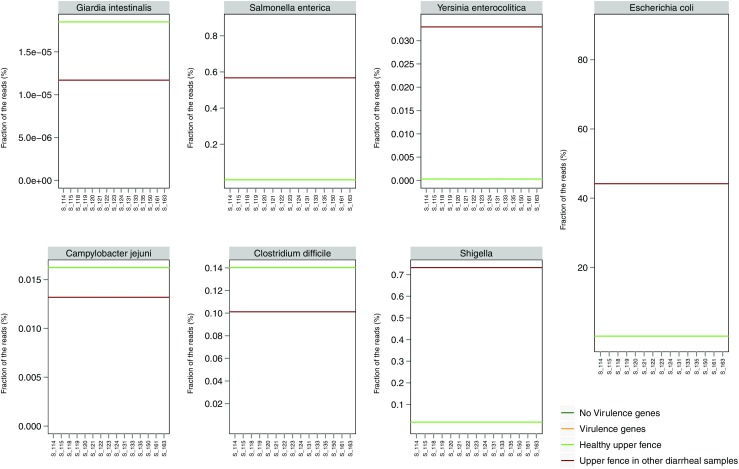
Relative abundance of pathogens in samples negative by conventional diagnostics. For samples that were either negative or virus-positive by conventional diagnostics, the fraction of reads mapping to each pathogen (*Giardia*, *Salmonella*, *Y. enterocolitica*, *E. coli*, *C. jejuni*, *C. difficile* and *Shigella*) was plotted. The *orange dots* indicate the presence of pathogen-specific virulence genes, while the *green dots* indicate the absence. The upper fence (Q_3_ + 1.5×IQR) of the relative abundance for healthy controls and for the diarrhoea samples where the particular pathogen was not detected by conventional methods are shown

Figure 3 shows the abundance of pathogens and the presence of virulence factors for the 11 sequenced conventionally negative samples and the four samples where only viral pathogens were determined by the conventional diagnostics. None of these sample contained *Giardia*, *C. difficile* or *Shigella*. One sample (S_120) was above the threshold in *Salmonella* relative abundance, whereas another (S_119) contained reads mapping to *Salmonella* virulence genes *invA* and *invE*. *Campylobacter* was detected in four samples, where virulence genes were detected in three negative samples (S_114, S_118, S_119) and one positive for norovirus (S_135).

For *E. coli*, four samples (S_118, S_119, S_121, S_135) were above the threshold and *E. coli* virulence gene *eae* was detected in two samples (S_135, S_163). Only one sample (S_120) was above the threshold for *Y. enterocolitica*, but no virulence genes were detected.

The analysis of negative samples using the ratio between the relative abundance and Shannon’s diversity index showed similar results (Supplemental Fig. 4).

### Comparison of results obtained from conventional diagnostics and direct sequencing of faecal samples

In Table 2, the similarities and discrepancies between the conventional findings and the NGS-based findings on faecal samples are listed, covering all samples included in the study. The NGS approach was able to identify pathogens in agreement with conventional diagnostics in 34 of the 38 samples (Table 2). Five of these samples (S_130, S_132, S_153, S_154 and S_164) were conventionally positive for two different bacterial pathogens, with correct identification of both by the NGS approach. However, for four samples (S_129, S_136, S_149 and S_158), neither abundance nor virulence factors indicated the presence of the pathogens identified by the conventional diagnostics (one *S. typhimurium*, two *C. difficile* and one ETEC).

**Table 2:** Similarities and discrepancies between the conventional and NGS-based diagnostics

	*Giardia*		*Salmonella*		*Y. enterocolitica*		*E. coli*		*Shigella*		*C. jejuni*		*C. difficile*		Sample(s)	
Conv. positive	Conv.	NGS	Conv.	NGS	Conv.	NGS	Conv.	NGS	Conv.	NGS	Conv.	NGS	Conv.	NGS	No.	ID
	+	+	–	–	–	–	–	–	–	–	–	–	–	–	2	S_111, S_155
	–	–	+	+	–	–	–	–	–	–	–	–	–	–	1	S_126*
	–	–	+	+	–	–	–	+	–	–	–	–	–	–	1	S_127
	–	–	+	+	–	–	–	–	–	–	–	–	–	+	1	S_128
	–	–	+	+	–	–	+	+	–	–	–	–	–	–	1	S_153
	–	–	+	–	–	–	–	–	–	–	–	–	–	–	1	S_129
	–	–	–	–	+	+	–	–	–	–	–	–	–	–	1	S_143
	–	–	–	–	+	+	–	–	–	–	–	–	+	+	1	S_164
	–	–	–	–	–	–	+	+	–	–	–	–	–	–	11	S_110, S_140, S_141, S_142, S_145, S_148*, S_151, S_154, S_156, S_157, S_159
	–	–	–	–	–	–	+	+	–	–	–	–	+	+	1	S_130*
	–	–	–	–	–	–	+	+	–	–	+	+	–	–	1	S_132*
	–	–	–	–	–	–	+	–	–	–	–	–	–	–	1	S_158
	–	–	–	–	–	–	–	–	–	–	–	–	+	+	9	S_105, S_106, S_107, S_134, S_137, S_160, S_162, S_165, S_166
	–	–	–	–	–	–	–	–	+	+	–	–	–	–	2	S_144, S_152
	–	–	–	–	–	–	–	–	–	–	+	+	–	–	4	S_102, S_103, S_104, S_138*
	–	–	–	–	–	–	–	–	–	–	–	–	+	–	2	S_136, S_149*
	–	NA	–	NA	–	NA	–	NA	–	NA	–	NA	+	NA	1	S_108
	–	–	–	+	–	–	–	+	–	–	–	+	–	–	1	S_135*
	–	–	–	–	–	–	–	–	–	–	–	–	–	–	3	S_131*, S_133*, S_150*
Conv. negative	–	–	–	–	–	–	–	–	–	–	–	–	–	–	6	S_115, S_121, S_122, S_123, S_124, S_161
	–	–	–	–	–	–	–	–	–	–	–	+	–	–	2	S_114, S_118
	–	NA	–	NA	–	NA	–	NA	–	NA	–	NA	–	NA	2	S_116, S_117
	–	–	–	+	–	+	–	–	–	–	–	–	–	–	1	S_120
	–	–	–	+	–	–	–	–	–	–	–	+	–	–	1	S_119
	–	–	–	–	–	–	–	+	–	–	–	–	–	–	1	S_163
	–	–	–	–	–	–	–	–	–	–	–	–	–	–	8	H_101, H_103, H_104, H_105, H_106, H_107, H_108, H_110
	+^a^	–	–	–	–	–	–	–	–	–	–	–	–	–	1	H_102
	–	–	–	–	–	–	–	–	–	–	–	+^b^	–	–	1	H_111

Samples with positive findings in either conventional (*Conv*.) diagnostics or by NGS are denotedwith a +. An asterisk (*) denotes samples that are virus-positive according to conventionalmethods. In the conventional negative set is included both diarrhoea samples and healthy controls

^a^H_102 was conventionally positive for *Giardia* only by PCR

^b^Four reads of *Campylobacter*virulence gene *flaA* were detected

The NGS approach, on the other hand, was able to detect additional bacterial pathogens in samples positive for other pathogens by conventional diagnostic testing and in samples that had been tested negative. Of the 38 conventionally positive bacterial diarrhoea samples, two (S_127, S_128) were found to contain additional pathogens by the NGS approach (Table 2). In the conventionally negative diarrhoea samples, pathogens were detected in five of the 11 sequenced samples, as well as in one of the conventionally virus-positive samples (Table 2).

In two conventionally positive samples (S_127, S_135), *E. coli* was identified as an additional pathogen by the detection of reads mapping to *E. coli* virulence gene *eae* encoding intimin. Similarly, in three other conventionally positive samples (S_104, S_107, S_129), reads mapping to the virulence gene *astA* were detected, and this virulence gene was also detected in one healthy control sample (H_108). However, the detection of this gene alone was considered insufficient for pathogen prediction, since *astA* can be present in healthy individuals [29].

*Campylobacter jejuni* was detected as an additional pathogen in one sample (S_135) that was conventionally positive for another pathogen and in three samples (S_114, S_118, S_119) considered negative. *Campylobacter jejuni* virulence gene *flaA* was detected in one healthy control sample (H_111). *Salmonella enterica* was detected as an additional pathogen in one sample (S_135) conventionally positive for another pathogen and in two negative samples (S_119, S_120), while *C. difficile* was not detected in any of the conventionally negative samples.

### Detection of isolate sequence within the metagenomic sample

**Fig. 4:**
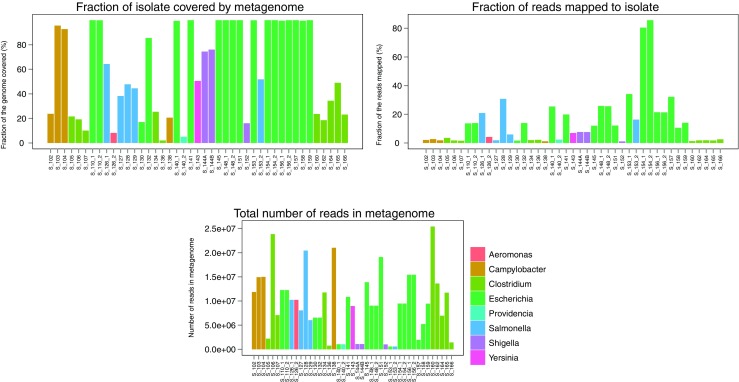
Isolate detection within metagenomic samples. Reference mapping of reads from the metagenomic sample against the isolate sequence from the specific sample was employed to assess the percentage of the isolate covered by the metagenomic sequencing. Also, the fraction of reads within the metagenomic sample that were used in mapping is illustrated, as well as the total number of reads present in the metagenomic sequences

For 35 of the 38 patient samples with bacterial findings by conventional diagnostics, the percentage of the isolate covered by reads in the metagenomic sample ranged between 2 and 100%, as illustrated in Fig. 4. Three samples were not included in this analysis due to unavailability of the isolate (S_137, S_149) and an isolate sequence file error (S_142).

### Detection of virulence genes

All virulence genes found in *E. coli* by conventional PCR were also detected by NGS on single isolates (Table 3), in addition to many more. Not all virulence genes found in the single isolates were observed in the metagenomics data.

**Table 3:** Virulence factors detected by PCR or sequencing of single isolates and by metagenomics

Sample	Pathogen	Virulence genes found by PCR*	Virulence genes found in isolate and metagenome	Missing in isolate	Missing in metagenome, but found in isolate
S_102	*C. jejuni*		*flaA*, *ciaB*, *cdtA*, *flaB*, *cdtB*, *flaC*, *cdtC*		*cdtC*
S_103	*C. jejuni*		*cdtB*, *flaB*, *flaC*, *flaA*, *cdtC*, *cdtA*, *ciaB*		
S_104	*C. jejuni*		*flaA*, *cdtC*, *ciaB*, *cdtA*, *cdtB*, *flaB*, *flaC*		
S_105	*C. difficile*		*cdtB*, *cdtA*, *tcdB*, *tcdA*		
S_106	*C. difficile*		*cdtB*, *tcdB*, *tcdA*		*cdtA*
S_107	*C. difficile*		*tcdB*, *tcdA*, *cdtB*		*cdtA*
S_110	*E. coli*	*eae*, *stx1A*	*eae*, *stx1B*, *stx1A*	*astA*	*stxB*, *stxA*
S_126	*S. enterica*		*invH*, *invE*, *invG*, *invA*, *sseE*, *sseC*, *invJ*, *invB*		*sseA*, *sseB*, *sseD*
S_127	*S. enterica*		*invG*		*invJ*, *invE*, *sseD*, *sseB*, *sseC*, *sseA*, *invA*, *sseE*, *invH*, *invB*
S_128	*S. enterica*		*invA*		*sseC*, *sseB*, *sseD*, *invE*, *invJ*, *invG*, *sseA*, *invB*, *invH*, *sseE*
S_129	*S. enterica*				*sseD*, *invJ*, *invE*, *sseC*, *sseB*, *invA*, *sseA*, *invG*, *invH*, *invB*, *sseE*
S_130	*E. coli*	*eae*		*eae*, *astA*	
S_132	*E. coli*	*stx1A*	*stx1A*	*eae*	*stx1B*, *stxA*, *stxB*
S_134	*C. difficile*		*tcdB*, *tcdA*, *cdtB*		*cdtA*
S_136	*C. difficile*				*tcdB*, *cdtA*, *tcdA*, *cdtB*
S_138	*C. jejuni*		*flaB*		*cdtB*, *cdtA*, *flaA*, *cdtC*, *ciaB*, *flaB*, *flaC*
S_140	*E. coli*	*eae*	*eae*		*eltA*
S_141	*E. coli*	*eae*		*eae*	
S_142	*E. coli*		*eltA*, *astA*, *eltB*		
S_143	*Y. enterocolitica*		*inv*		*ystB*
S_144	*Shigella*		*ipaH7.8*, *ipaH9.8*, *mxiA*, *virA*, *ipaB*		*ipaD*, *ipaC*
S_145	*E. coli*	*astA*, *eltA*	*eltB*, *astA*, *eltA*		
S_148	*E. coli*	*eae*	*eae*		
S_151	*E. coli*	*astA*	*astA*	*eae*, *eltB*, *aggR*	
S_152	*Shigella*		*ipaH7.8*, *virA*, *ipaB*, *ipaH9.8*, *mxiA*, *ipaD*		*ipaC*
S_153	*E. coli*	*eae*	*eae*		
S_153	*S. enterica*		*sseB*		*sseD*, *invE*, *invJ*, *sseC*, *invA*, *invG*, *sseA*, *invB*, *invH*, *sseE*
S_154	*E. coli*	*aggR*	*aggR*	*eae*, *astA*	
S_156	*E. coli*	*eae*	*astA*, *eae*	*sta*, *aggR*	
S_157	*E. coli*	*astA*, *eltA*	*astA*, *eltB*, *eltA*		
S_158	*E. coli*	*astA*			*astA*
S_159	*E. coli*	*astA*, *eltA*	*eltB*, *astA*, *eltA*	*eae*	
S_160	*C. difficile*		*cdtB*, *tcdA*, *tcdB*		*cdtA*
S_162	*C. difficile*		*tcdA*, *cdtB*		*cdtA*, *tcdB*
S_164	*C. difficile*		*tcdB*, *tcdA*, *cdtB*		*cdtA*
S_165	*C. difficile*		*cdtB*, *tcdB*, *cdtA*, *tcdA*		
S_166	*C. difficile*		*tcdA*, *cdtA*, *tcdB*, *cdtB*		

*Only for *E. coli*

### Typing of *E. coli* and *C. difficile* by direct sequencing

**Fig. 5:**
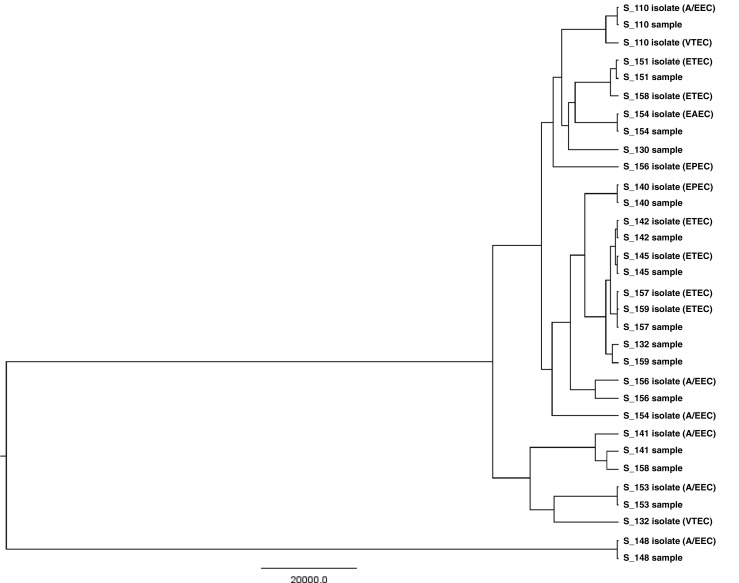
Phylogenetic relationships among metagenomic samples and isolates. An NDtree is shown for *E. coli*. The tree was constructed by mapping isolate WGS sequences and complete metagenomic sequences against the reference *E. coli* O157:H7 str. Sakai. *Escherichia coli* pathotypes are shown in parentheses on isolates

A phylogenetic tree for *E. coli*, including the metagenomic sequences and isolate sequences, is illustrated in Fig. 5. The tree shows clear matches between most metagenomic samples and their respective isolates. For *C. difficile*, the data output was very low, with only 2–49% of the *C. difficile* genomes being represented in the metagenomic samples, and, thus, it was not found to be sufficient for the construction of a meaningful phylogenetic tree.

## Discussion

Accurate and rapid diagnostics of human pathogens are important in order to direct treatment of infectious disease and prevent possible outbreaks. Faeces is a complex material containing high numbers of different bacteria, different clones within the same species, commensals and potential pathogens. Thus, an NGS diagnostic approach, as with any other diagnostic approach, requires the ability to detect the presence of potential pathogens as well as the ability to differentiate between potential pathogens and their commensal variants. This is a complicated process as some species, e.g. *E. coli*, can be both, and since healthy persons can sometimes be asymptomatic carriers of otherwise pathogenic bacteria. Therefore, the interpretation of test results needs to take patient symptoms and previous treatment, as well as knowledge of the epidemiology of pathogens, into account. This study showed that direct sequencing of faecal samples is a new method for identifying faecal pathogens from patients with diarrhoea. We found that the NGS approach was able to identify the majority of bacterial pathogens identified by conventional diagnostic methods (34 of 38 samples). The NGS approach further detected bacterial pathogens that were probably responsible for the diarrhoeal disease in five of the 11 clinical samples in which no pathogens could be detected by conventional diagnostics and in four samples where other pathogens had been detected. This has the potential to affect patient management. Additionally, the NGS approach was able to assign the correct pathotype for the diarrhoeagenic *E. coli* by detection of the pathotype-specific virulence genes. In this pilot study, we used a threshold based on the upper fence abundance observed in diarrhoeal samples and controls, respectively, together with the detection of specific virulence genes. This approach might be too sensitive and possibly associated with false-positive samples, potentially leading to unnecessary treatment. Thus, before implementing this shotgun metagenomic approach in the clinical setting, further studies are required, which enable a proper definition of the threshold for relative abundance and the detection of virulence genes (i.e. number of reads needed, percentage of the gene covered). Furthermore, when using the conventional identification as criteria, there is a risk that mistakes might include positive samples in the control group. As the number of samples increases, this is, however, expected to cause limited problems. Ideally, future studies should be carried out under field conditions, i.e. in clinical settings, and lead to a definition of a threshold corresponding to where treatment leads to cure of the patient.

*Campylobacter jejuni* was the most common pathogen detected in the samples from patients with diarrhoea found to be negative by conventional methods. *Campylobacter* are known to be difficult to culture, and it has previously been found that PCR methods were able to detect *Campylobacter* species in culture-negative samples, probably as a result of decreased viability of the cells [30]. Although uncultivable, *Campylobacter* DNA would have been detectable by direct sequencing of the samples and it is, therefore, reasonable to assume that the conventionally (culture) negative samples were from *Campylobacter* infections. *Salmonella* was also found more often by the NGS approach than by culturing. The identification of *Salmonella* in the laboratory is based on colony identification on enteric medium, and recognition requires skilled personnel. Therefore, *Salmonella* may be under-diagnosed by conventional methods, but reduced viability, as for *C. jejuni*, may also play a role.

*Escherichia coli* was also found more often by NGS than by conventional diagnostics, and because *E. coli* is also a commensal, the NGS detection relied solely on well-known virulence genes, where specifically *eae* was found, which is also targeted by PCR in the conventional diagnostics. However, as *E. coli* is abundant in the samples, probably with several strains simultaneously, one explanation for the discrepancy could be that the *eae*-containing strain was never the one to be subjected to PCR in the conventional diagnostic.

For most of the pathogens examined, the relative abundance worked well for the case definition of a proportion of the samples, while for some samples, the detection of pathogen-specific virulence genes was necessary for identifying the pathogen. For both *E. coli* and *Shigella*, the relative abundance of the bacteria alone was not considered sufficient for a positive finding by our method, as sequences specific for these pathogens were just as abundant among negative samples as those that were conventionally positive. *Shigella* is not a commensal, but from a taxonomic perspective, it is a member of the *E. coli* species [31]. Thus, sequence similarities between *Shigella* and *E. coli* could be distorting the picture. However, the detection of *Shigella*-specific and *E. coli*-specific virulence genes worked well.

Limitations of the NGS approach for diagnostics relates to the extent of the databases used for mapping, since only pathogens included in the databases can be detected. This may explain why six samples which were negative by conventional methods remained negative by NGS analysis, where only *C. jejuni*, *S. enterica*, *E. coli*, *Shigella*, *C. difficile* and *Giardia* were specifically targeted. Thus, it is probable that other diarrhoeagenic organisms could have been missed. However, the patients could have also suffered from non-infectious diarrhoea. The NGS method can be further improved in the future by expansion of the databases to include more diarrhoeagenic pathogens and specific targets.

The inability to detect the responsible bacterial pathogen by our method in four of the conventionally positive samples was most likely a result of too little sequence data. For *C. difficile*, this might be caused by the DNA purification method used, which might not be optimal for DNA obtained from Gram-positive species. Thus, in future studies, an optimised procedure should be employed [32]. Also, the large variation in the amount of pathogen sequences detected in the samples, and the percentage of the bacterial genomes covered by reads of the metagenomic sequencing, reflected the variations in the sequence output. For future analyses, these types of samples should be sequenced deeper to ensure obtainment of enough sequence data to properly detect the responsible pathogen, despite the pathogen often constituting only a small fraction of the DNA purified from the samples. Specifically, there is a huge variation in the amount of human DNA present, as well as some of the potential pathogens, e.g. *C. difficile* and *C. jejuni* are present in very low abundance. More sequence data should be obtained per sample in future analyses, which should also evaluate larger sets of samples and target more pathogens.

Our NGS approach did not allow for detection of the viral pathogens that had been found by conventional diagnostic testing, as most of the viral pathogens were RNA viruses, which were not targeted by the DNA purification method employed in our study. However, the NGS approach could be expanded to include RNA purification from stool samples and construction of specific databases for virus diagnostics. Furthermore, parasite detection was inadequate, as only a few complete parasite genome sequences were available to be referenced in MGmapper. However, we did construct a custom database of parasite draft genomes for the detection of *Giardia* sequences, and further development of this database will improve the diagnosis of parasite infections.

In addition to providing pathogen identification, direct sequencing can also offer extensive additional information on, for instance, virulence and resistance genes, or the presence of other pathogens, e.g. fungi and DNA viruses, which can be extracted from the data. Also, non-culturable pathogens can potentially be identified. Our NGS approach showed the potential of performing bacterial typing from direct sequencing, as *E. coli*-positive samples showed almost perfect phylogenetic clustering with their corresponding isolates. We did not investigate whether it was possible to infer phylogenies when only including metagenomics data, but the results suggest that metagenomics data may, as a minimum, be used in combination with single isolates for more rapid elucidation and tracking of outbreaks. Typing could probably be improved for both pathogens by obtaining more sequences per sample.

The findings in this study indicate a future value of direct sequencing of clinical faecal samples for diagnostic purposes. This method may still be considered too labourious and expensive for routine use in clinical settings where tens of thousands of samples have to be processed annually. However, the costs associated with the current conventional diagnostics and failure to identify the causative agent in a large part of the samples should not be ignored. Furthermore, as prices and turnaround times for NGS are declining, this type of analysis may become available in first-line diagnostic setups offering rapid diagnostics and providing valuable information to help direct patient treatment. No estimates of turnaround time or costs associated with conventional diagnostics or our meta-genomic approach was calculated. However, for single targets, i.e. isolates, we recently compared WGS and the conventional approach for surveillance of VTEC and found WGS to be both cheaper and faster [17]. Further studies also including economical calculations, including those associated with delayed diagnostics and increased morbidity and mortality, are called for.

## Electronic supplementary material

Below are the links to the electronic supplementary material.

Supplemental File 1 Additional information on “Materials and methods” (DOCX 19 kb)

Supplemental Fig. 1 Relative abundance of pathogens in healthy control samples (EPS 28 kb)

Supplemental Fig. 2 Heat map of the most abundant bacterial genera. The heat map shows the relative abundance of the top 20 most abundant genera among the samples, and Pearson correlation is used for clustering. The blue colour gradient illustrates the relative abundance of the genus in the sample, while white colour fields are not among the 20 most abundant in the specific sample. On the coloured bars at the top, samples are coloured according to the pathogen(s) detected by the conventional methods (EPS 655 kb)

Supplemental Fig. 3 Ratio between the relative abundance of pathogens in samples positive by conventional diagnostics and Shannon’s diversity index of the sample. For each pathogen (*Giardia*, *Salmonella*, *Y. enterocolitica*, *E. coli*, *C. jejuni*, *C. difficile* and *Shigella*), the fraction of reads mapping to the pathogen divided by the Shannon diversity index is plotted for all samples positive by the conventional diagnostic methods. The *orange dots* indicate the presence of pathogen-specific virulence genes as determined by NGS analysis, while the *green dots* indicate the absence. The upper fence (Q3 + 1.5×IQR) of the relative abundance for the healthy controls and for the diarrhoea samples where the particular pathogen was not detected by conventional methods are shown (EPS 2716 kb)

Supplemental Fig. 4 Ratio between the relative abundance of pathogens in samples negative by conventional diagnostics and Shannon’s diversity index of the sample. For samples that were either negative or virus-positive by conventional diagnostics, the fraction of reads mapping to each pathogen (*Giardia*, *Salmonella*, *Y. enterocolitica*, *E. coli*, *C. jejuni*, *C. difficile* and *Shigella*) divided by the Shannon diversity index is plotted. The *orange dots* indicate the presence of pathogen-specific virulence genes, while the *green dots* indicate the absence. The upper fence (Q3 + 1.5×IQR) of the relative abundance for healthy controls and for diarrhoea samples where the particular pathogen was not detected by conventional methods are shown (EPS 2755 kb)

ESM 1 (DOCX 17 kb)
