# Supplementary material for: Evaluating next-generation sequencing for direct clinical diagnostics in diarrhoeal disease
Source: Eur J Clin Microbiol Infect Dis. 2017 Mar 11;36(7):1325–38. doi: 10.1007/s10096-017-2947-2 (PMC5495851; doi:10.1007/s10096-017-2947-2)
Supplement: Supplementary file 1 — Additional information on “Materials and methods” (DOCX 19 kb) [file 10096_2017_2947_MOESM1_ESM.docx]

1. Species confirmation and virulence profiling of isolate sequences

The *KmerFinder* performs species prediction based on the number shared k-mers (16-mers) between the query genome and a reference database, where only those k-mers with prefix ATGAC were stored, to reduce the number of *k-*mers. The reference database consisted of 2,787 complete bacterial genomes retrieved from NCBI. For each isolate sequence, species prediction was made on basis of the complete genome in the reference database with most 16-mer hits.

1. Metagenomic Analysis and Species Distribution in faecal samples

Briefly, the MGmapper software consists of three main steps: pre-processing of raw reads, mapping of reads to the reference sequence databases, and post-processing of mapping results. The pre-processing step is done using cutadapt [1], which performs common adapter removal and trims the low-quality ends from reads (cutoff value of 30) and later discards reads that are shorter than 30 bp. In the second step, already trimmed reads are aligned to a pre-defined set of reference sequence databases using bwa mem [2]. Samtools [3] are used to remove singletons and filter reads where neither a read nor its mate is mapped. Reads can be mapped in either fullmode or bestmode. In fullmode, all the trimmed reads are considered in the alignment. In bestmode, mapping is performed against all specified reference databases, simultaneously. Subsequently, for each read pair the best hit among all alignments is chosen. A pair of reads is considered as a hit only if the sum of the alignment scores (SAS) is higher than any SAS values from other database hits. If a pair of reads has identical SAS values when mapping to several databases, the only one pair, associated with the database that was specified first in the list of reference databases, is kept. In the last, post-processing step, alignments are filtered based on matches/mis-matches threshold, which is specified as fraction or absolute number. In this analysis, the default matches/mis-matches threshold of 80% is used. The *Virulence Factor* and *VirulenceFinder* databases were mapped in fullmode; and the other four databases were mapped in bestmode, with the databases in following order: complete bacterial genomes, draft bacterial genomes, human genomes, and parasites. The MGmapper is a pipeline facilitating the abovementioned steps from user-defined parameters, whereas the visualization of the output for comparison among samples was performed through manual bioinformatics analysis. Specifically, mapping information from the MGmapper tool was visualized using ggplot2 plotting system for R [4].

[1] Martin M. Cutadapt removes adapter sequences from high-throughput sequencing reads. EMBnet J 2011;17:10–2.

[2] Li H, Durbin R. Fast and accurate long-read alignment with Burrows-Wheeler transform. Bioinformatics 2010;26:589–95. doi:10.1093/bioinformatics/btp698.

[3] Li H, Handsaker B, Wysoker A, Fennell T, Ruan J, Homer N, et al. The Sequence Alignment/Map format and SAMtools. Bioinformatics 2009;25:2078–9. doi:10.1093/bioinformatics/btp352.

[4] Wickham H. ggplot2: Elegant Graphics for Data Analysis. Springer; 2009.
