# Supplementary material for: Evaluating next-generation sequencing for direct clinical diagnostics in diarrhoeal disease
Source: Eur J Clin Microbiol Infect Dis. 2017 Mar 11;36(7):1325–38. doi: 10.1007/s10096-017-2947-2 (PMC5495851; doi:10.1007/s10096-017-2947-2)
Supplement: Supplementary file 6 — (DOCX 17 kb) [file 10096_2017_2947_MOESM6_ESM.docx]

Supplementary table 1 Virulence factors detected by PCR or sequencing of single isolates and by metagenomics

| **Sample** | **Pathogen** | **Virulence genes found by PCR*** | **Virulence genes found in isolate and metagenome** | **Missing in isolate** | **Missing in metagenome, but found in isolate** |
| --- | --- | --- | --- | --- | --- |
| S_102 | C. jejuni |  | flaA,ciaB,cdtA,flaB,cdtB,flaC,CdtC |  | cdtC |
| S_103 | C. jejuni |  | cdtB,flaB,flaC,flaA,cdtC,cdtA,ciaB |  |  |
| S_104 | C. jejuni |  | flaA,cdtC,ciaB,cdtA,cdtB,flaB,flaC |  |  |
| S_105 | C. difficile |  | cdtB,cdtA,tcdB,tcdA |  |  |
| S_106 | C. difficile |  | cdtB,tcdB,tcdA |  | cdtA |
| S_107 | C. difficile |  | tcdB,tcdA,cdtB |  | cdtA |
| S_110 | E. coli | eae,stx1A | eae,stx1B,stx1A | astA | stxB,stxA |
| S_126 | S. enterica |  | invH,invE,invG,invA,sseE,sseC,invJ,invB |  | sseA,sseB,sseD |
| S_127 | S. enterica |  | invG |  | invJ,invE,sseD,sseB,sseC,sseA,invA,sseE,invH,invB |
| S_128 | S. enterica |  | invA |  | sseC,sseB,sseD,invE,invJ,invG,sseA,invB,invH,sseE |
| S_129 | S. enterica |  |  |  | sseD,invJ,invE,sseC,sseB,invA,sseA,invG,invH,invB,sseE |
| S_130 | E. coli | eae |  | eae,astA |  |
| S_132 | E. coli | Stx1A | stx1A | eae | stx1B,stxA,stxB |
| S_134 | C. difficile |  | tcdB,tcdA,cdtB |  | cdtA |
| S_136 | C. difficile |  |  |  | tcdB,cdtA,tcdA,cdtB |
| S_138 | C. jejuni |  | flaB |  | cdtB,cdtA,flaA,cdtC,ciaB,flaB,flaC |
| S_140 | E. coli | eae | eae |  | eltA |
| S_141 | E. coli | eae |  | eae |  |
| S_142 | E. coli |  | eltA,astA,eltB |  |  |
| S_143 | Y. enterocolitica |  | inv |  | ystB |
| S_144 | Shigella |  | ipaH7.8,ipaH9.8,mxiA,virA,ipaB |  | ipaD,ipaC |
| S_145 | E. coli | astA,eltA | eltB,astA,eltA |  |  |
| S_148 | E. coli | eae | eae |  |  |
| S_151 | E. coli | astA | astA | eae,eltB,aggR |  |
| S_152 | Shigella |  | ipaH7.8,virA,ipaB,ipaH9.8,mxiA,ipaD |  | ipaC |
| S_153 | E. coli | eae | eae |  |  |
| S_153 | S. enterica |  | sseB |  | sseD,invE,invJ,sseC,invA,invG,sseA,invB,invH,sseE |
| S_154 | E. coli | aggR | aggR | eae,astA |  |
| S_156 | E. coli | eae | astA,eae | sta,aggR |  |
| S_157 | E. coli | astA,eltA | astA,eltB,eltA |  |  |
| S_158 | E. coli | astA |  |  | astA |
| S_159 | E. coli | astA,eltA | eltB,astA,eltA | eae |  |
| S_160 | C. difficile |  | cdtB,tcdA,tcdB |  | cdtA |
| S_162 | C. difficile |  | tcdA,cdtB |  | cdtA,tcdB |
| S_164 | C. difficile |  | tcdB,tcdA,cdtB |  | cdtA |
| S_165 | C. difficile |  | cdtB,tcdB,cdtA,tcdA |  |  |
| S_166 | C. difficile |  | tcdA,cdtA,tcdB,cdtB |  |  |

*: only for *Escherichia coli*
